# Cross-neutralization of SARS-CoV-2 Kappa and Delta variants by inactivated vaccine-elicited serum and monoclonal antibodies

**DOI:** 10.1038/s41421-021-00347-1

**Published:** 2021-11-23

**Authors:** Lin Cheng, Shuo Song, Qing Fan, Senlin Shen, Haiyan Wang, Bing Zhou, Xiangyang Ge, Bin Ju, Zheng Zhang

**Affiliations:** 1 Institute for Hepatology, National Clinical Research Center for Infectious Disease, Shenzhen Third People’s Hospital; The Second Affiliated Hospital, School of Medicine, Southern University of Science and Technology, Shenzhen, Guangdong China; 2 Guangdong Key Laboratory for Anti-Infection Drug Quality Evaluation, Shenzhen, Guangdong China; 3 Shenzhen Research Center for Communicable Disease Diagnosis and Treatment of Chinese Academy of Medical Science, Shenzhen, Guangdong China

**Keywords:** Immunology, Molecular biology

Dear Editor,

The SARS-CoV-2 variant of B.1.617 lineage was first identified in India in October 2020, and rapidly spread to many other countries around the world. The variant includes three main sub-lineages, B.1.617.1 (Kappa), B.1.617.2 (Delta), and B.1.617.3^1^. In light of their high transmissibility, the Kappa and Delta variants had been designated as a variant of interest and a variant of concern by the World Health Organization, respectively. As shown in Fig. 1a, the two variants shared L452R mutation in the receptor-binding domain (RBD). In addition, the variants also harbor unique mutations: E484Q in the Kappa variant and T478K in the Delta variant^2^.

**Fig. 1:**
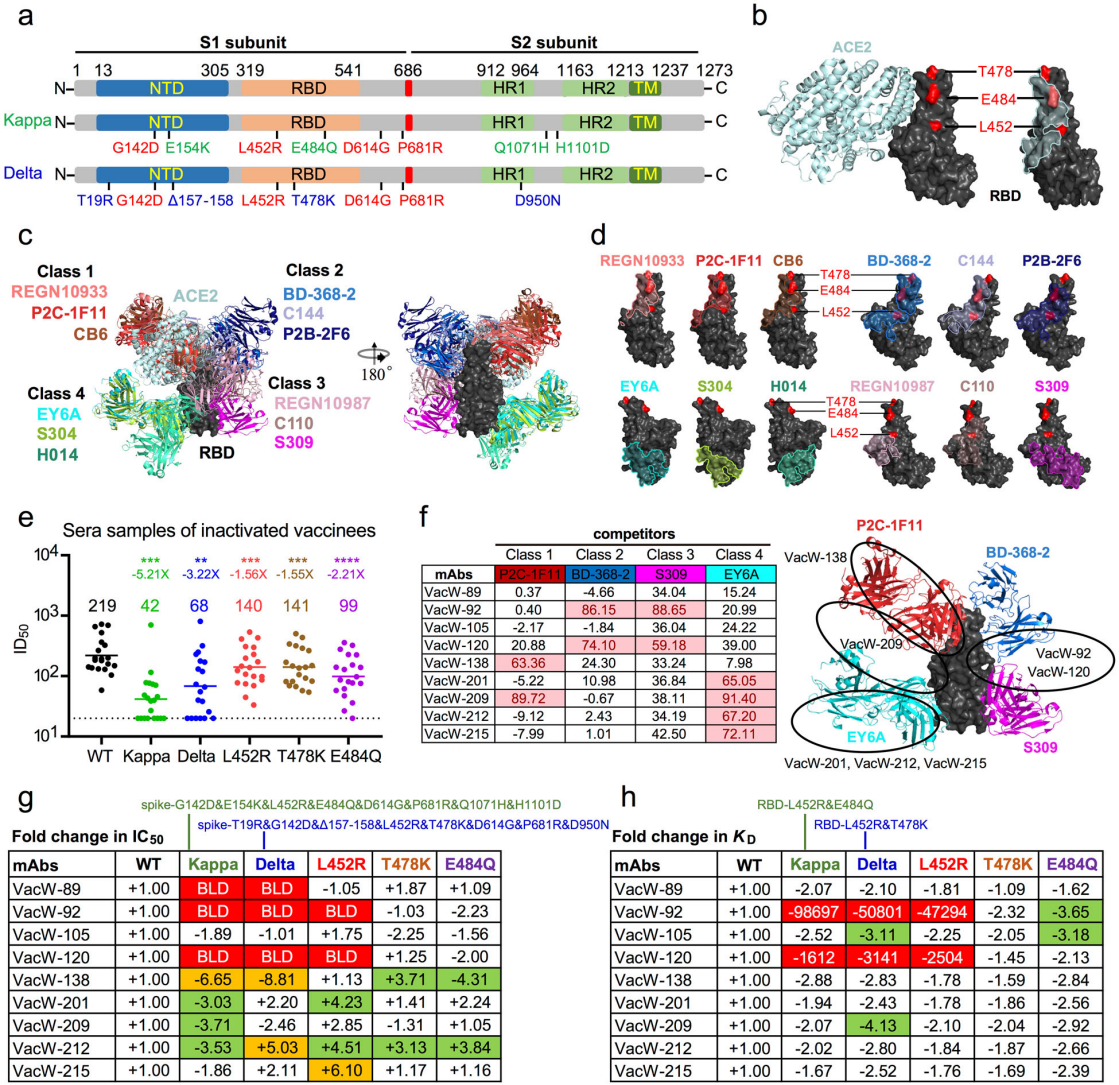
Neutralization of SARS-CoV-2 Kappa and Delta variants by inactivated vaccine-elicited serum and monoclonal antibodies. **a** Mutations located in the viral spike proteins were identified in the Kappa and Delta variants. Shared mutations in both were highlighted in red, unique mutations in Kappa were marked in green, and mutations in Delta were in blue. **b** Overall structure of ACE2 in complex with SARS-CoV-2 RBD (left) and footprint of ACE2 on the RBD (right). Three mutation residues (L452, T478, and E484) were shown in red on the RBD. **c** Structural depiction of ACE2 and representative nAbs from each class binding to the RBD. Class 1: P2C-1F11, REGN10933, CB6; Class 2: BD-368-2, C144, P2B-2F6; Class 3: S309, C110, REGN10987; Class 4: EY6A, S304, H014. **d** Footprints of four classes of representative nAbs on the RBD. Three mutation residues (L452, T478, and E484) were shown in red on the RBD. **e** Changes in serum neutralizing titers of inactivated vaccine recipients against Kappa and Delta, as well as L452R, T478K, and E484Q variants, compared with WT. GMTs in ID_50_ values were calculated and shown above each variant. **P < 0.01, ***P < 0.001, ****P < 0.0001. **f** Competition ELISA (left) and predicted recognizing epitopes (right) of tested monoclonal nAbs binding to RBD with four representative nAbs. The values of competition measured in the highest concentration (10 μg/mL) over 50% were highlighted in light red. **g** The fold change in IC_50_ values of each monoclonal nAbs against mutant and WT viruses. **h** The fold change in affinity values in binding capacities of each nAb to mutant and WT RBD proteins. Data shown in **e**–**h** were means of two or three independent experiments. The changes shown in **g**, **h** between 3-fold and 5-fold were marked in green, between 5-fold and 10-fold were in orange. Below the limit of detection (BLD) in red indicated that the inhibition of nAbs was < 50% even in the highest concentration (50 μg/mL), and changes in affinities more than 10-fold were also highlighted in red. Symbol ‘+’ indicates increased neutralization or affinity, and ‘–’ indicates decreased neutralization or affinity.

SARS-CoV-2 utilized the RBD of viral spike protein to recognize its cellular receptor (angiotensin-converting enzyme 2, ACE2), and disruption of the RBD–ACE2 interaction could block virus entry, which was usually regarded as the candidate target of antiviral drugs and neutralizing antibodies (nAbs)^3^. Therefore, mutations that appeared in RBD may affect the viral infection and transmission, and may even lead to the escape of SARS-CoV-2 from the neutralization of nAbs elicited by natural virus infection and induced by vaccine^4^. The three mutation residues (L452, T478, and E484) were involved in or near the footprint of ACE2 on the RBD (Fig. 1b), which may influence the binding affinity of ACE2 and neutralizing activity of nAbs.

It is widely known that RBD-specific nAbs could be divided into four classes according to the competition with ACE2 and the accessibility of recognizing epitopes on the RBD in ‘up’ or ‘down’ conformation (Fig. 1c)^3^. The three mutations (L452R, E484Q, and T478K) that appeared in Kappa and Delta variants were located in and near the binding interface between RBD and most of the nAbs from Classes 1, 2, and 3, indicating that these two variants may escape from the neutralization of nAbs. It was encouraging that nAbs of Class 4 recognized a distinct epitope away from the mutation region of SARS-CoV-2 Kappa and Delta variants, which may still retain neutralizing activities (Fig. 1d).

Nowadays, various SARS-CoV-2 vaccines, including inactivated, mRNA, DNA, adenovirus vector, and recombination protein vaccines, have been developed to combat the viral infection^5^. The immunogenicity of vaccines usually varied in different platforms with distinct amounts of antigen and stimulation times. Even the same type of vaccines from different companies induced varying degrees of immune responses^6^. Although a recent small-sample study reported that the geometric mean titer (GMT) of neutralizing antibodies induced by the inactivated vaccine (Coronavac) was about tenfold lower than that induced by the mRNA vaccine (BNT162b2)^7^, inactivated vaccines still showed good immunogenicity and protective effect against SARS-CoV-2 in animal models and clinical trials^8,9^. Meanwhile, several studies have encouragingly revealed that despite a certain degree of decline, vaccinated sera preserved neutralizing activities against Alpha and Beta variants^10^. However, little is known about the protective efficacy of the inactivated vaccine against Kappa and Delta variants. Especially, the cross-neutralization of monoclonal nAbs isolated from inactivated vaccine recipients has yet to be reported.

Here, we prepared a series of SARS-CoV-2 pseudoviruses bearing spike proteins of the Wuhan reference strain (wild type, WT), Kappa, or Delta variants, as well as L452R, E484Q, or T478K single mutations based on the HIV-1 backbone, which is widely accepted to evaluate the neutralizing activities of various serum samples and monoclonal nAbs^11,12^. Twenty vaccinated serum samples with high values of RBD-specific binding antibodies collected at about 2 weeks after immunization with two doses of inactivated vaccine were incorporated into this study (Supplementary Table S1). We first measured the neutralizing activities of these serum samples against WT, Kappa, and Delta variants (Supplementary Fig. S1). The levels of neutralizing antibodies against Kappa and Delta variants were lower than those against WT, which significantly displayed a 5.21-fold reduction (42 vs 219) and a 3.22-fold decline (68 vs 219) respectively in GMTs (Fig. 1e and Supplementary Fig. S2a). We further evaluated the influence of three single mutations present in RBD of variants on their escape from polyclonal nAbs, and the neutralizing activities of serum samples against L452R, T478K, and E484Q variants were reduced by 1.56-, 1.55-, and 2.21-fold compared to those against WT (Fig. 1e and Supplementary Fig. S2a). These data indicated that SARS-CoV-2 Kappa and Delta variants reduced their sensitivities to the inactivated vaccine-elicited serum to some extent. In addition, the neutralizing potency of serum against variants was strongly related to that against WT strain (Supplementary Fig. S2b).

Two groups have reported the neutralizing activities of monoclonal nAbs isolated from volunteers who received mRNA or inactivated vaccines, suggesting that vaccines could effectively induce nAbs against WT SARS-CoV-2 and several variants^12,13^. However, it is poorly understood whether Kappa and Delta variants escape from the neutralization of monoclonal nAbs elicited by inactivated vaccine. Here, we used the SARS-CoV-2 RBD protein as a bait to sort specific single B cells by flow cytometry from two volunteers (Supplementary Fig. S3). Nine monoclonal nAbs were isolated and characterized, whose neutralizing potencies ranged from 0.03 to 11.11 μg/mL against WT (Supplementary Figs. S4 and S6a). The gene usages of these nAbs were derived from multiple germlines including IGHV1–46, 1–69, 3–23, 3–7, etc. (Supplementary Table S2). Importantly, the heavy chains of VacW-209 and VacW-215 belonged to IGHV3−30 and 3–53 germlines, respectively, which were also termed as public antibodies and usually shared among COVID-19 patients^14,15^. More encouragingly, VacW-209 was a potent neutralizer with IC_50_ of 0.03 μg/mL similar to that of P2C-1F11 identified from a COVID-19 patient, the Fc-modified version (named Brii-196) of which is being investigated in phase 3 of the clinical trial. In addition, the neutralizing activity of VacW-209 was more potent than previously reported nAbs isolated from inactivated vaccinees^13^.

Using competition ELISA, we predicted the binding epitopes of these nine nAbs based on their competition with four representative nAbs. As shown in Fig. 1f and Supplementary Fig. S5, VacW-92 and VacW-120 were classified into Class 2/3, VacW-138 recognized the epitope of Class 1, VacW-201, VacW-212, and VacW-215 belonged to Class 4 nAbs, and VacW-209 exhibited high competition with both P2C-1F11 (Class 1) and EY6A (Class 4) of binding to RBD. We failed to predict the recognizing epitopes of VacW-89 and VacW-105 due to their low binding affinities. It was also possible that these two nAbs might recognize some novel epitopes beyond the four classes described above. Overall, these results demonstrated that the inactivated vaccine induced a similar RBD-specific antibody response to natural SARS-CoV-2 infection.

Finally, we further tested the cross-neutralizing and binding activities of these nine nAbs against SARS-CoV-2 Kappa and Delta variants, as well as several single mutations (Supplementary Figs. S4 and S6a). Most of the nAbs still maintained their neutralizing activities despite some with slight reductions, except for VacW-89, VacW-92, and VacW-120, whose inhibitions were below 50% when tested at the highest concentration (50 μg/mL) (Fig. 1g). Of note, VacW-209 effectively neutralized all tested variants, including Kappa, Delta, L452R, T478K, and E484Q, with similar potencies to that of the above-mentioned P2C-1F11 (Supplementary Fig. S4). The neutralizing activities of monoclonal nAbs decreased with the same trend as those of the two vaccinee serum samples, with obvious reductions for Kappa and Delta variants and moderate alterations for single point mutations. Consistently, the binding affinities of these nAbs to WT and mutant RBD proteins (RBD-WT, L452R&E484Q, L452R&T478K, L452R, T478K, and E484Q) displayed the same pattern with their neutralizing activities (Fig. 1h and Supplementary Figs. S6b, S7 and Table S3), indicating a strong correlation between binding avidity and neutralization. VacW-92 and VacW-120 bound very weakly to the mutant RBD proteins bearing L452R, which revealed the escape mechanism of SARS-CoV-2 variants from the neutralization of antibodies.

In conclusion, we comprehensively reported the neutralization of serum antibodies elicited by the inactivated vaccine against SARS-CoV-2 Kappa and Delta variants. The cross-neutralization of serum samples against mutant viruses partially decreased compared with that against WT, and the mutation E484Q resulted in a more significant decline of neutralizing activity than L452R and T478K. These findings were consistent with other studies showing similar loss of neutralization of serum samples from convalescent patients or induced by mRNA or adenovirus vector vaccines^1,2^. Although it does exist a reduction in the neutralization of inactivated vaccine-elicited sera, they really maintained some degree of protective immunity against the infection of mutant viruses, especially the Delta variant, which is still raging across the world and causes more serious disease processes. Encouragingly, we isolated and characterized a series of monoclonal nAbs from inactivated vaccine recipients, whose germline gene usages, recognition of epitopes, neutralizing potencies, and cross-neutralization against SARS-CoV-2 variants were similar to those nAbs elicited by natural virus infection. Taken together, our results emphasized the importance of the immunization of inactivated vaccine to prevent infection of Kappa and Delta variants, which could contribute to controlling the pandemic. We have also proved the ability of the inactivated vaccine to induce broadly monoclonal nAbs and identified a potent neutralizer VacW-209 which could serve as a candidate therapeutic antibody in eliciting passive protection against various SARS-CoV-2 variants.

## Supplementary information

supplementary information
