# Supplementary material for: Cross-neutralization of SARS-CoV-2 Kappa and Delta variants by inactivated vaccine-elicited serum and monoclonal antibodies
Source: Cell Discov. 2021 Nov 23;7:112. doi: 10.1038/s41421-021-00347-1 (PMC8608826; doi:10.1038/s41421-021-00347-1)
Supplement: Supplementary file 1 — supplementary information [file 41421_2021_347_MOESM1_ESM.pdf]

Supplementary Information

Materials and Methods

Study approval and biological samples

This study was approved by the Ethics Committee of Shenzhen Third People's Hospital, China (approval number: 2020-030). All participants had provided written informed consent for sample collection and subsequent analysis. All serum and PBMC samples from individuals received two doses of inactivated SARS-CoV-2 vaccines (WIBP-CorV, the Sinopharm COVID-19 vaccine, Wuhan Institute of Biological Products Co., Ltd) were collected at about 2 weeks after the second vaccination from the Shenzhen Third People's Hospital. All serum samples were stored at -80°C and heat-inactivated at 56°C for 1h before use. Serum IgG and IgM values of binding antibodies to the SARS-CoV-2 RBD were measured using the Chemiluminescence immunoassay kit[1]. PBMCs were maintained in freezing medium and stored in liquid nitrogen.

SARS-CoV-2 pseudovirus-based neutralizing assay

SARS-CoV-2 pseudovirus was generated by co-transfection of HEK-293T cells with 10 μg of SARS-CoV-2 spike-expressing plasmid and 20 μg of an env-deficient HIV-1 backbone vector (pNL4-3.Luc.R-E-). Two days post-transfection, the culture supernatant was harvested, clarified by centrifugation, filtered and stored at -80°C. To determine the neutralizing activity, serially diluted serum samples or monoclonal antibodies were incubated with equal volume of diluted pseudovirus at 37°C for 1h. The HEK-293T-hACE2 cells were subsequently added in duplicate. After a 48h incubation, the culture medium was removed and 100 μl of the Bright-Lite Luciferase reagent (Vazyme Biotech) was added to the cells. After a 2-min incubation at RT, 90 μl of cell lysate was transferred to 96-well white solid plates for measurements of luminescence using the Varioskan™ LUX multimode microplate reader (Thermo Fisher Scientific). The 50% inhibitory dilution (ID_{50}) or 50% inhibitory concentration (IC_{50}) was calculated using GraphPad Prism 8.0 software by log (inhibitor) vs. normalized response - Variable slope (four parameters) model.

Isolation of monoclonal antibodies from inactivated vaccinees

Thawed PBMCs were stained with an antibody cocktail consisting of CD19-PE-

Cy7, CD3-Pacific Blue, CD8-Pacific Blue, CD14-Pacific Blue, and IgG-FITC (all from BD Biosciences) to gate IgG+ B cells. SARS-CoV-2 WT RBD with His tag (Sino Biological) was used as a probe to sort antigen-specific single B cells. Two anti-His secondary antibodies labeled with APC and PE (Abcam) were both used to recognize the RBD bait and exclude the nonspecific staining. Flow cytometric data was acquired on Aria II flow cytometer (BD Biosciences) and analyzed using FlowJo software (TreeStar). Single B cells were sorted into 96-well PCR plates containing lysis buffer followed by RT-PCR and nested PCR to amplify variable regions of heavy and light chains, respectively. Variable genes were sequenced, synthesized, and separately cloned into the full-length IgG1 heavy and light chain expression vectors. Monoclonal antibodies were expressed by co-transfection of 293F cells with paired heavy and light chain plasmids and purified from the culture supernatants using protein A column.

Enzyme linked immunosorbent assay (ELISA) and competition ELISA

SARS-CoV-2 RBD protein was coated into 96-well plates at 4°C overnight. The plates were washed with PBST buffer and blocked with 5% skim milk and 2% bovine albumin in PBS at RT for 1h. Serially diluted monoclonal antibodies were added into wells and incubated at 37°C for 1h. The plates were washed and HRP conjugated goat anti human IgG antibodies (ZSGB-BIO) were added and then incubated at 37°C for 1h. Finally, the TMB substrate (BD Biosciences) was added and incubated at RT for 20mins and the reaction was stopped by 2M H_2SO_4. The readout was detected at a wave length of 450nm. For competition ELISA, four representative competitor nAbs (P2C-1F11, BD-368-2, S309, and EY6A) coupled with HRP were mixed with serially diluted testing antibodies, added into the plates, and then incubated at 37°C for 1h. The following steps were the same as the standard ELISA mentioned above. The testing antibodies were 3-fold serially diluted from 10 μg/ml, and the antibody concentration in the last dilution was as low as 0.005 μg/ml which was considered no competition. The percentage of competition was calculated by the formula: (1-OD_{450}/OD_{450} in the last dilution) × 100%, and 50% was set as the cutoff suggesting an obvious competition. The results of four representative nAbs competing with themselves demonstrated this formula could correctly predict the epitopes recognized by testing antibodies.

Binding analysis by surface plasmon resonance (SPR)

The binding assays of monoclonal antibodies to the wild type and mutant SARS-CoV-2 RBD proteins (Sino Biological) were performed using the Biacore 8K system (GE Healthcare). Specifically, one flow cell of the CM5 sensor chips were covalently coated with the wild type or mutant RBDs (Sino Biological) in 10 mM sodium acetate buffer (pH 5.0) for a final RU (response units) around 250, whereas the other flow cell was left uncoated and blocked as a control. All the assays were run at a flow rate of 30 µl/min in HBS-EP buffer (10 mM HEPES pH 7.4, 150 mM NaCl, 3 mM EDTA, and 0.05% Tween-20). Serially diluted antibodies were injected for 60s respectively and the resulting data were fit in a 1:1 binding model with Biacore Evaluation software (GE Healthcare). Every measurement was performed two times and the individual values were used to produce the mean affinity constant.

Statistical analysis

Statistical analysis was performed with paired t test using GraphPad Prism 8.0 software. Symbol '**' means P < 0.01, '***' means P < 0.001, '****' means P < 0.0001.

Protein Data Bank (PDB) code

ACE2 (PDB code: 7DMU), P2C-1F11 (PDB code: 7CDI), REGN10933 (PDB code: 6XDG), CB6 (PDB code: 7C01), BD-368-2 (PDB code: 7CHH), C144 (PDB code: 7K90), P2B-2F6 (PDB code: 7BWJ), S309 (PDB code: 6WPS), C110 (PDB code: 7K8V), REGN10987 (PDB code: 6XDG), EY6A (PDB code: 6ZCZ), S304 (PDB code: 7JW0), H014 (PDB code: 7CAI).

References

1 Yu, S. *et al.* Distinct kinetics of immunoglobulin isotypes reveal early diagnosis and disease severity of COVID-19: A 6-month follow-up. *Clin Transl Med* **11**, e342, doi:10.1002/ctm2.342 (2021).

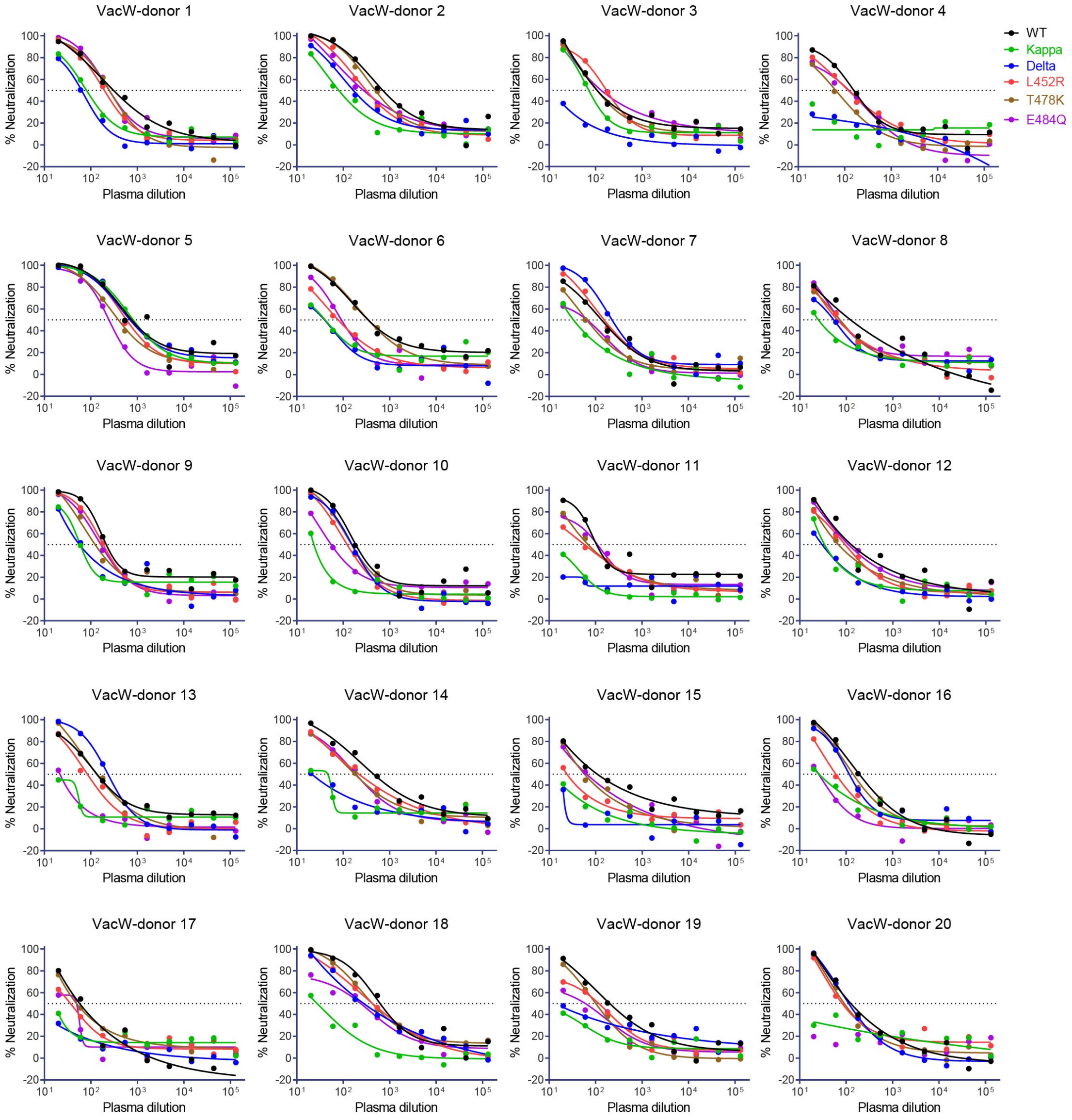

Supplementary Figure S1. The neutralization of each inactivated vaccinee serum sample against SARS-CoV-2 variants.

One out of two or three independent experiments with similar results.

a

ID50	WT	Kappa	Delta	L452R	T478K	E484Q
VacW-donor 1	364.90	73.59	65.77	307.28	317.58	278.50
VacW-donor 2	723.51	77.77	177.33	410.43	434.34	224.16
VacW-donor 3	158.70	76.56	< 20	172.68	92.63	139.82
VacW-donor 4	193.54	< 20	< 20	123.50	69.24	138.86
VacW-donor 5	658.08	700.59	806.96	535.52	340.18	322.69
VacW-donor 6	325.26	48.23	39.94	77.41	288.98	97.28
VacW-donor 7	147.99	43.23	249.34	197.81	82.60	61.65
VacW-donor 8	131.59	45.69	55.15	86.43	81.46	76.93
VacW-donor 9	264.72	115.03	73.10	228.29	139.67	152.43
VacW-donor 10	180.24	39.59	170.16	145.80	149.43	57.68
VacW-donor 11	129.06	< 20	< 20	74.23	66.89	86.52
VacW-donor 12	183.67	70.62	38.12	107.26	81.13	128.80
VacW-donor 13	141.64	< 20	230.32	99.23	135.88	26.55
VacW-donor 14	524.74	38.64	25.70	298.72	264.24	227.10
VacW-donor 15	103.88	< 20	< 20	33.14	57.26	75.47
VacW-donor 16	199.55	26.84	119.20	69.29	169.09	36.01
VacW-donor 17	58.52	< 20	< 20	44.44	54.22	47.21
VacW-donor 18	692.12	< 20	317.42	430.32	503.15	357.01
VacW-donor 19	199.12	< 20	< 20	138.49	146.45	80.76
VacW-donor 20	136.86	< 20	130.83	97.90	105.37	< 20

Fold change	WT	Kappa	Delta	L452R	T478K	E484Q
VacW-donor 1	+1.00	-4.96	-5.55	-1.19	-1.15	-1.31
VacW-donor 2	+1.00	-9.30	-4.08	-1.76	-1.67	-3.23
VacW-donor 3	+1.00	-2.07	BDL	+1.09	-1.71	-1.14
VacW-donor 4	+1.00	BDL	BDL	-1.57	-2.80	-1.39
VacW-donor 5	+1.00	+1.06	+1.23	-1.23	-1.93	-2.04
VacW-donor 6	+1.00	-6.74	-8.14	-4.20	-1.13	-3.34
VacW-donor 7	+1.00	-3.42	+1.68	+1.34	-1.79	-2.40
VacW-donor 8	+1.00	-2.88	-2.39	-1.52	-1.62	-1.71
VacW-donor 9	+1.00	-2.30	-3.62	-1.16	-1.90	-1.74
VacW-donor 10	+1.00	-4.55	-1.06	-1.24	-1.21	-3.12
VacW-donor 11	+1.00	BDL	BDL	-1.74	-1.93	-1.49
VacW-donor 12	+1.00	-2.60	-4.82	-1.71	-2.26	-1.43
VacW-donor 13	+1.00	BDL	+1.63	-1.43	-1.04	-5.33
VacW-donor 14	+1.00	-13.58	-20.41	-1.76	-1.99	-2.31
VacW-donor 15	+1.00	BDL	BDL	-3.13	-1.81	-1.38
VacW-donor 16	+1.00	-7.43	-1.67	-2.88	-1.18	-5.54
VacW-donor 17	+1.00	BDL	BDL	-1.32	-1.08	-1.24
VacW-donor 18	+1.00	BDL	-2.18	-1.61	-1.38	-1.94
VacW-donor 19	+1.00	BDL	BDL	-1.44	-1.36	-2.47
VacW-donor 20	+1.00	BDL	-1.05	-1.40	-1.30	BDL

b

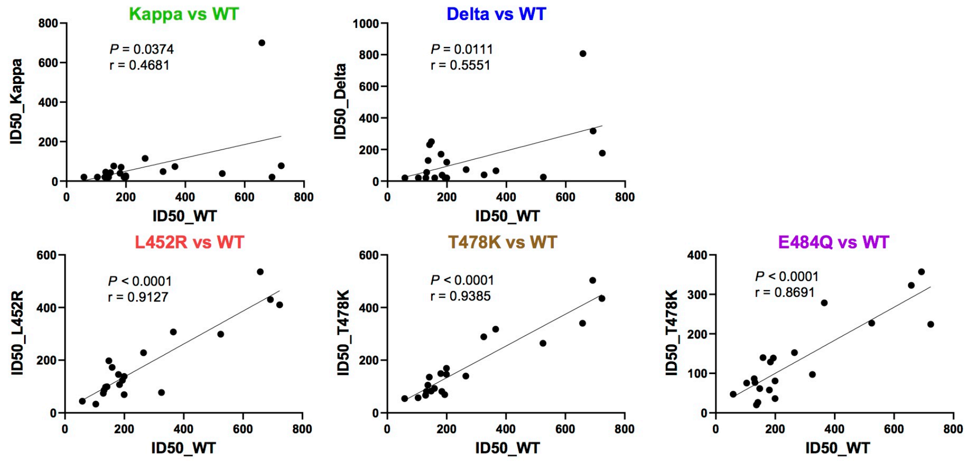

Supplementary Figure S2. The neutralization and fold change of each inactivated vaccinee serum sample against SARS-CoV-2 variants.

(a) The ID_{50} values (left) and fold changes (right) in neutralizations of each serum sample against mutated and WT viruses. The data was mean of two or three independent experiments. Neutralizing resistances increased between 3-fold and 5-fold were highlighted in green, those between 5-fold and 10-fold were in orange, those more than 10-fold were in red. Below the detection limit (BDL) also in red indicated that the inhibition of serum sample was less than 50% even in the highest concentration (1:20 dilution). Symbol '+' indicates increased sensitivity, '-' indicates increased resistance. **(b)** Correlation analysis between the values of ID_{50} against variants (Kappa, Delta, L452R, T478K, and E484Q) and WT virus. $P < 0.05$ was considered significant.

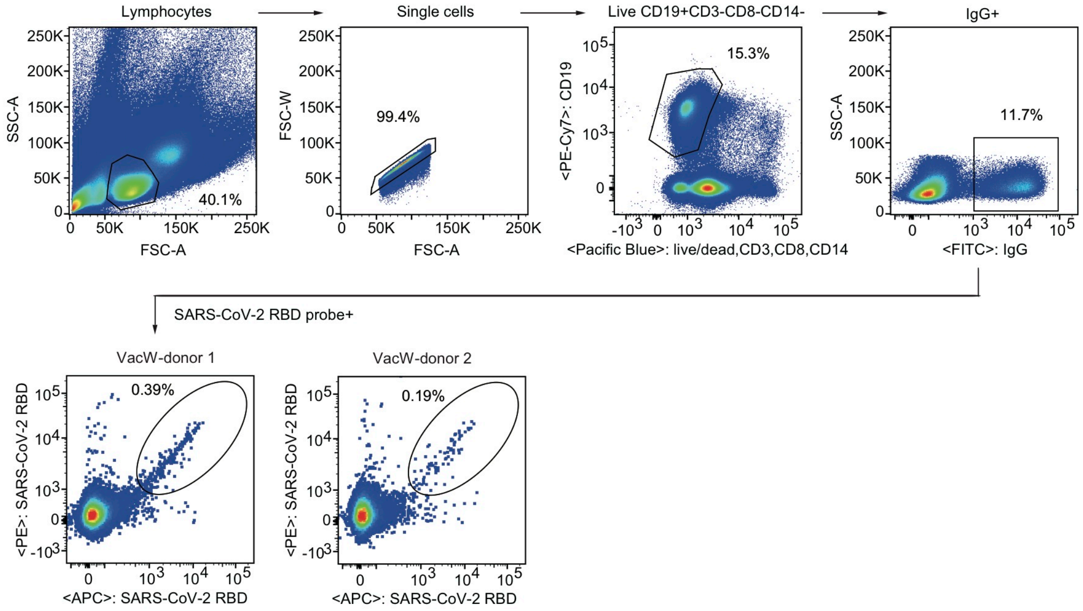

Supplementary Figure S3. The gating strategy for isolation of SARS-CoV-2 WT RBD-specific B cells by FACS.

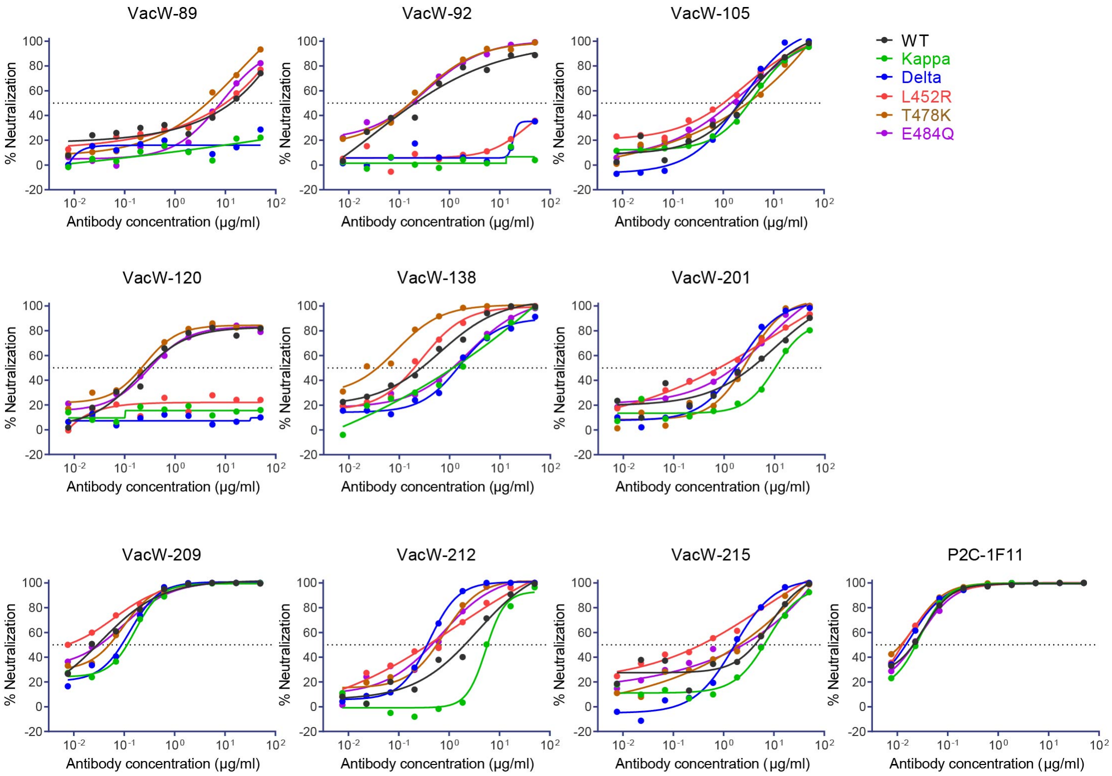

Supplementary Figure S4. The neutralization of each monoclonal nAb against SARS-CoV-2 variants.

P2C-1F11 was a positive control. One out of two independent experiments with similar results.

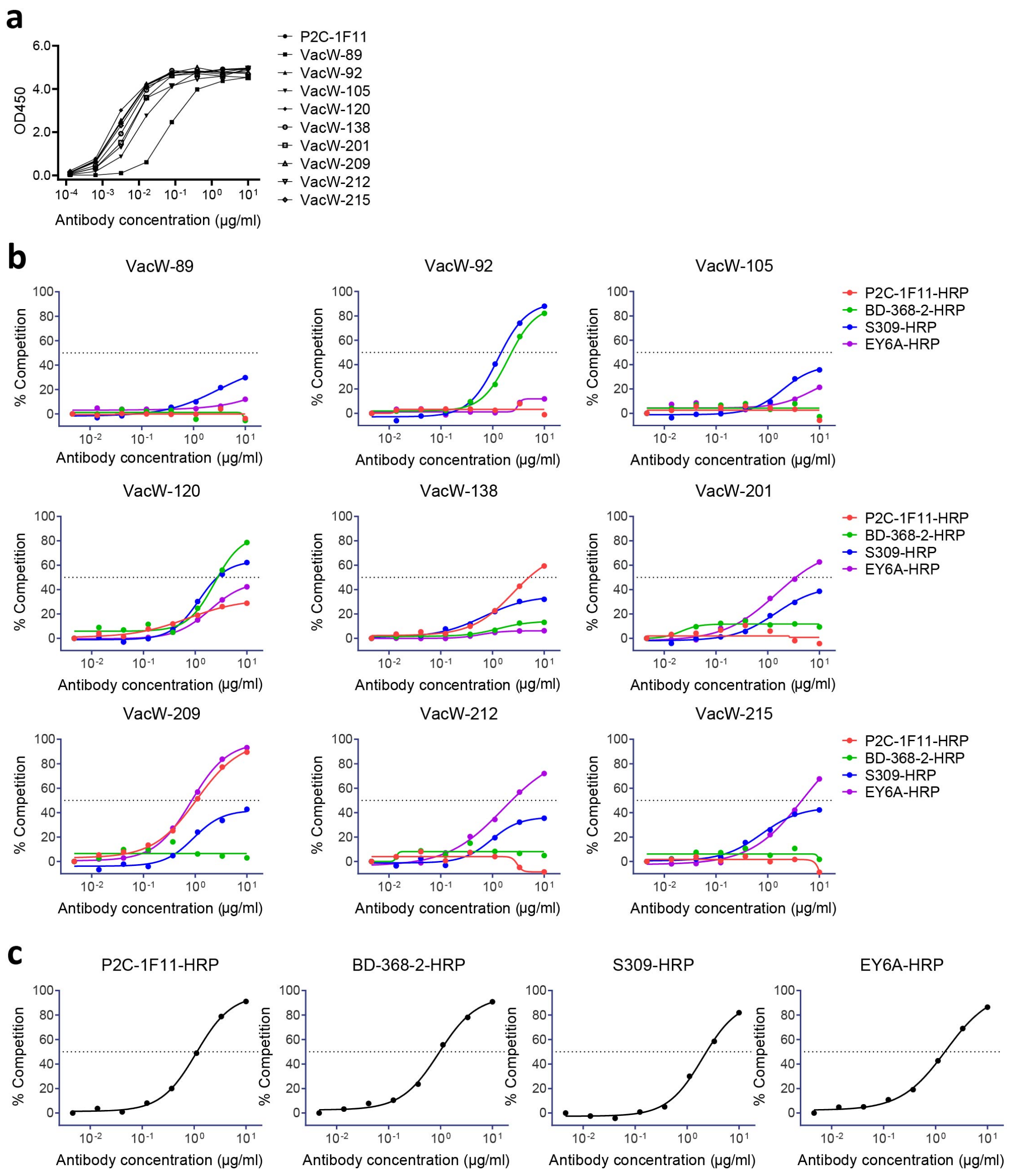

Supplementary Figure S5. ELISA and competition ELISA of monoclonal nAbs isolated from inactivated vaccinees binding to SARS-CoV-2 RBD. **(a)** ELISA binding of nine nAbs to the SARS-CoV-2 WT RBD protein. P2C-1F11 was a positive control. **(b)** Competition ELISA of tested nAbs with representative nAbs of four classes. Class 1: P2C-1F11-HRP, Class 2: BD-368-2-HRP, Class 3: S309-HRP, Class 4: EY6A-HRP. **(c)** Competition ELISA of four representative nAbs with themselves served as standard controls. **(b-c)** A cutoff of 50% was indicated by a horizontal dashed line. One out of two independent experiments with similar results.

a

spike-G142D&E154K&L452R&E484Q&D614G&P681R&Q1071H&H1101D

spike-T19R&G142D&Δ157-158&L452R&T478K&D614G&P681R&D950N

IC50 (ug/ml)	WT	Kappa	Delta	L452R	T478K	E484Q
VacW-89	11.11	>50	>50	11.66	5.95	10.22
VacW-92	0.17	>50	>50	>50	0.18	0.38
VacW-105	1.82	3.44	1.83	1.04	4.09	2.83
VacW-120	0.26	>50	>50	>50	0.21	0.53
VacW-138	0.26	1.73	2.29	0.23	0.07	1.12
VacW-201	3.38	10.22	1.54	0.80	2.39	1.51
VacW-209	0.03	0.11	0.07	0.01	0.04	0.03
VacW-212	1.56	5.50	0.31	0.35	0.50	0.41
VacW-215	3.11	5.79	1.47	0.51	2.65	2.69

b

RBD-L452R&E484Q

RBD-L452R&T478K

KD (nM)	WT	Kappa	Delta	L452R	T478K	E484Q
VacW-89	3.10	6.41	6.51	5.62	3.39	5.02
VacW-92	0.0099	985	507	472	0.0232	0.0364
VacW-105	1.64	4.13	5.10	3.69	3.37	5.22
VacW-120	0.0503	81.1	158	126	0.0731	0.107
VacW-138	1.10	3.17	3.11	1.96	1.75	3.12
VacW-201	0.880	1.71	2.14	1.57	1.64	2.25
VacW-209	0.0113	0.0234	0.0467	0.0237	0.0230	0.0330
VacW-212	0.653	1.32	1.83	1.20	1.22	1.74
VacW-215	0.405	0.676	1.02	0.714	0.683	0.967

Supplementary Figure S6. The neutralizing and binding activities of each monoclonal nAb against SARS-CoV-2 variants.

(a) The IC_{50} values in neutralizations of each monoclonal nAbs against mutated and WT viruses. **(b)** The affinity values in binding capacities of each nAbs to mutated and WT RBD proteins. These data shown were means of two independent experiments. The changes between 3-fold and 5-fold were marked in green, between 5-fold and 10-fold were in orange. Below the detection limit (>50) in red indicated that the inhibition of nAbs was less than 50% even in the highest concentration (50 μg/ml). The changes in affinities more than 10-fold were also highlighted in red.

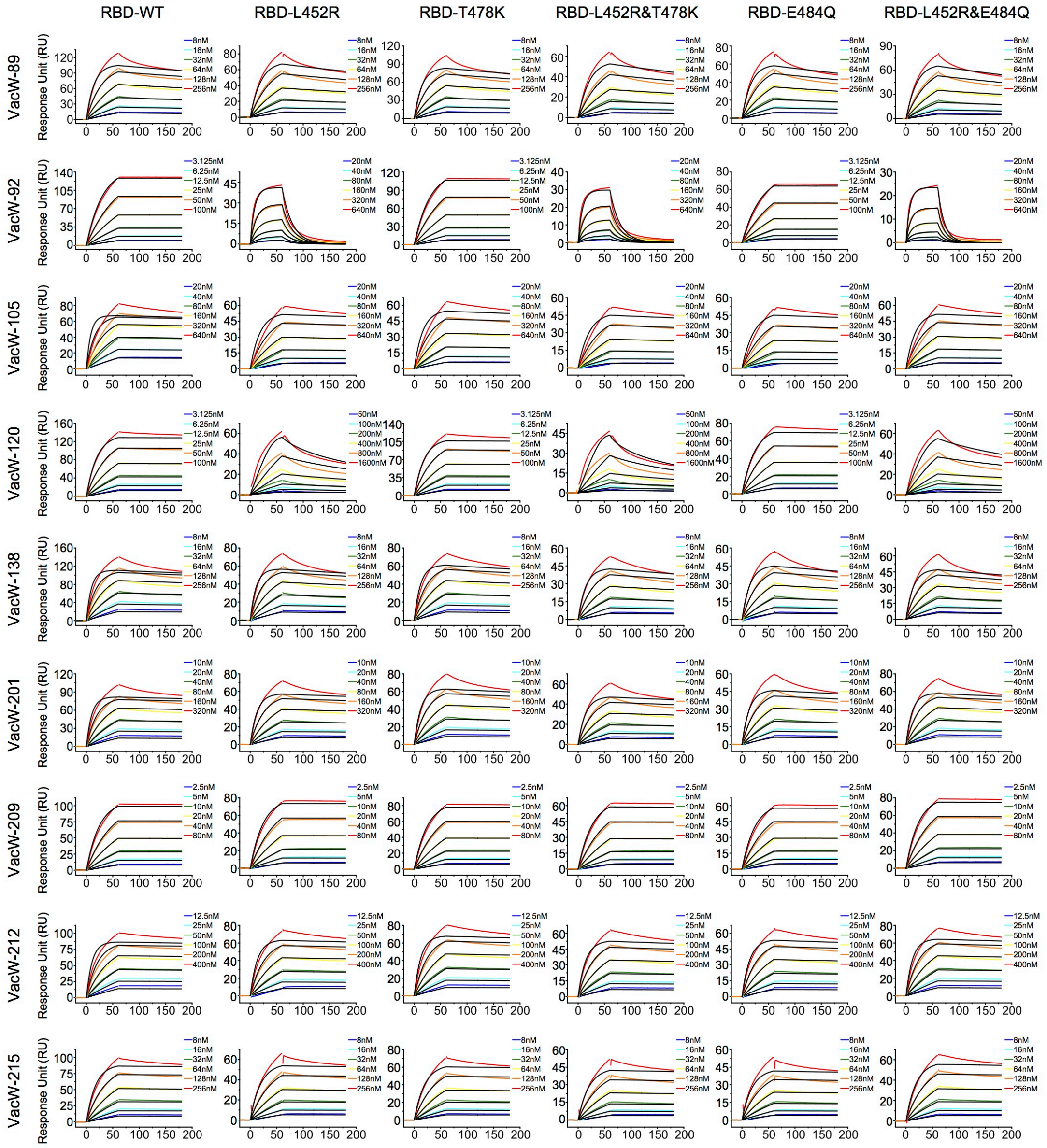

Supplementary Figure S7. The binding affinities of each monoclonal nAb to SARS-CoV-2 WT and mutated RBD proteins measured by SPR.

One out of two independent experiments with similar results.

Supplementary Table S1. The information of study subjects and their serum IgG and IgM binding values to SARS-CoV-2 RBD.

Vaccinees	Gender	Age	First vaccination	Second vaccination	Blood sampling	IgG	IgM
VacW-donor 1	Male	30	2020/12/9	2020/12/31	2021/1/11	19.04	0.96
VacW-donor 2	Female	36	2020/12/10	2020/12/31	2021/1/14	18.07	0.42
VacW-donor 3	Male	33	2020/12/10	2020/12/31	2021/1/13	17.08	0.91
VacW-donor 4	Female	22	2020/12/9	2020/12/31	2021/1/11	16.78	0.54
VacW-donor 5	Female	24	2020/12/15	2020/12/31	2021/1/13	16.49	3.17
VacW-donor 6	Female	34	2020/12/9	2020/12/31	2021/1/12	14.88	0.95
VacW-donor 7	Female	27	2020/12/10	2020/12/31	2021/1/12	14.83	0.85
VacW-donor 8	Female	35	2020/12/10	2020/12/31	2021/1/12	14.48	0.71
VacW-donor 9	Male	29	2020/12/9	2020/12/31	2021/1/14	14.42	0.30
VacW-donor 10	Female	26	2020/12/9	2020/12/31	2021/1/13	13.61	0.98
VacW-donor 11	Female	24	2020/12/10	2020/12/31	2021/1/11	13.59	1.37
VacW-donor 12	Female	24	2020/12/10	2020/12/31	2021/1/12	13.41	3.41
VacW-donor 13	Female	22	2020/12/9	2020/12/31	2021/1/13	13.23	0.17
VacW-donor 14	Male	31	2020/12/10	2020/12/31	2021/1/11	12.58	0.95
VacW-donor 15	Female	29	2020/12/9	2020/12/31	2021/1/12	12.55	2.17
VacW-donor 16	Female	23	2020/12/10	2020/12/31	2021/1/13	12.41	4.99
VacW-donor 17	Female	24	2020/12/10	2020/12/31	2021/1/11	11.81	0.17
VacW-donor 18	Female	26	2020/12/10	2020/12/31	2021/1/14	11.57	0.57
VacW-donor 19	Female	28	2020/12/9	2020/12/31	2021/1/14	11.35	0.64
VacW-donor 20	Female	43	2020/12/9	2020/12/31	2021/1/12	11.14	1.15

Supplementary Table S2. The gene family analysis of monoclonal nAbs isolated from two inactivated vaccinees.

Vaccinees	mAbs	Heavy chain					Light chain (Kappa or Lambda)			
		IGHV	IGHJ	IGHD	CDR3 length	SHM %	IGK(L)V	IGK(L)J	CDR3 length	SHM %
VacW-donor 1	VacW-92	1-46*01[a]	6*02	3-10*01	22	1.74	L7-46*01	L1*01	9	0.37
VacW-donor 1	VacW-105	1-69*09	5*02	2-21*02	16	1.74	L1-44*01	L1*01	11	0.75
VacW-donor 1	VacW-120	1-69*09	3*02	3-22*01	20	0.35	L1-40*01	L3*02	11	1.48
VacW-donor 1	VacW-89	3-23*04	4*02	1-1*01	13	0.00	K1-5*01	K3*01	9	0.00
VacW-donor 1	VacW-138	3-23*04	6*02	6-6*01	13	0.35	K1-13*02[c]	K3*01	9	1.14
VacW-donor 2	VacW-209	3-30*04[b]	6*02	3-22*01	24	3.12	L1-40*01	L3*02	11	0.00
VacW-donor 2	VacW-215	3-53*01	4*02	4-23*01	12	0.00	L6-57*02	L1*01	11	0.00
VacW-donor 2	VacW-201	3-7*01	4*02	4-23*01	12	0.69	L6-57*02	L3*02	11	0.00
VacW-donor 2	VacW-212	3-7*01	4*02	4-23*01	12	1.39	L6-57*02	L3*02	11	0.00

The program IMGT/V-QUEST was applied to analyze gene germline, complementarity determining region (CDR) 3 length, and somatic hypermutation (SHM). The CDR3 length was calculated from amino acids sequences. The SHM frequency was calculated from the mutated nucleotides.

[a]The heavy chain of VacW-92 was derived from IGHV1-46*01 or 1-46*03.

[b]The heavy chain of VacW-209 was derived from IGHV3-30*04 or 3-30-3*03.

[c]The light chain of VacW-138 was derived from IGKV1-13*02 or 1D-13*01 or 1D-13*02.

Supplementary Table S3. The summary of binding affinities of monoclonal nAbs to SARS-CoV-2 WT and mutated RBD proteins.

mAbs	RBD-WT			RBD-L452R			RBD-T478K			RBD-L452R&T478K			RBD-E484Q			RBD-L452R&E484Q		
	Ka(M-1·s-1)	Kd(s-1)	KD(nM)	Ka(M-1·s−1)	Kd(s-1)	KD(nM)	Ka(M-1·s−1)	Kd(s-1)	KD(nM)	Ka(M-1·s−1)	Kd(s-1)	KD(nM)	Ka(M-1·s−1)	Kd(s-1)	KD(nM)	Ka(M-1·s−1)	Kd(s-1)	KD(nM)
VacW-89	2.69E+05	8.45E-04	3.14	2.20E+05	1.25E-03	5.69	2.73E+05	9.34E-04	3.42	2.28E+05	1.48E-03	6.52	2.58E+05	1.31E-03	5.09	2.42E+05	1.56E-03	6.43
	2.73E+05	8.33E-04	3.05	2.31E+05	1.28E-03	5.55	2.75E+05	9.24E-04	3.36	2.36E+05	1.53E-03	6.49	2.67E+05	1.32E-03	4.95	2.53E+05	1.62E-03	6.39
VacW-92	3.29E+05	3.78E-06	0.0115	1.09E+05	5.15E-02	473	3.43E+05	7.69E-06	0.0224	1.07E+05	5.30E-02	496	2.79E+05	1.23E-05	0.0442	8.52E+04	8.09E-02	949
	3.30E+05	2.78E-06	0.00845	1.10E+05	5.16E-02	471	3.50E+05	8.36E-06	0.0239	1.06E+05	5.49E-02	517	2.91E+05	8.29E-06	0.0285	8.16E+04	8.28E-02	1020
VacW-105	1.90E+05	2.51E-04	1.32	8.62E+04	3.20E-04	3.71	9.67E+04	3.27E-04	3.38	7.63E+04	4.23E-04	5.54	7.13E+04	3.96E-04	5.55	8.97E+04	3.89E-04	4.34
	1.30E+05	2.55E-04	1.96	7.80E+04	2.87E-04	3.67	8.46E+04	2.83E-04	3.35	7.35E+04	3.42E-04	4.65	7.05E+04	3.44E-04	4.89	8.21E+04	3.21E-04	3.91
VacW-120	5.05E+05	2.54E-05	0.0504	1.08E+05	1.35E-02	125	5.37E+05	3.82E-05	0.0712	3.21E+05	5.26E-02	164	4.26E+05	4.44E-05	0.104	5.33E+04	4.34E-03	81.5
	5.07E+05	2.55E-05	0.0502	9.77E+04	1.23E-02	126	5.38E+05	4.03E-05	0.0749	2.14E+05	3.23E-02	151	4.23E+05	4.59E-05	0.109	5.28E+04	4.26E-03	80.6
VacW-138	4.20E+05	4.41E-04	1.05	3.60E+05	6.87E-04	1.91	3.34E+05	5.69E-04	1.70	2.73E+05	8.56E-04	3.13	2.65E+05	8.11E-04	3.06	2.87E+05	9.07E-04	3.16
	3.89E+05	4.49E-04	1.15	3.45E+05	6.88E-04	2.00	3.22E+05	5.82E-04	1.80	2.72E+05	8.39E-04	3.08	2.61E+05	8.27E-04	3.17	2.84E+05	9.00E-04	3.17
VacW-201	3.11E+05	2.74E-04	0.880	2.46E+05	3.81E-04	1.55	2.55E+05	4.22E-04	1.65	2.20E+05	4.77E-04	2.16	2.32E+05	5.19E-04	2.24	2.67E+05	4.59E-04	1.72
	3.04E+05	2.68E-04	0.879	2.44E+05	3.87E-04	1.59	2.55E+05	4.16E-04	1.63	2.19E+05	4.65E-04	2.12	2.31E+05	5.20E-04	2.26	2.66E+05	4.52E-04	1.70
VacW-209	5.06E+05	5.95E-06	0.0117	5.17E+05	1.14E-05	0.0221	5.09E+05	1.21E-05	0.0238	4.79E+05	2.28E-05	0.0476	5.22E+05	1.79E-05	0.0342	5.33E+05	1.29E-05	0.0242
	5.11E+05	5.58E-06	0.0109	5.18E+05	1.31E-05	0.0253	5.32E+05	1.18E-05	0.0222	4.82E+05	2.20E-05	0.0458	5.32E+05	1.69E-05	0.0318	5.40E+05	1.22E-05	0.0226
VacW-212	2.34E+05	1.48E-04	0.631	1.91E+05	2.24E-04	1.17	2.01E+05	2.41E-04	1.20	1.77E+05	3.22E-04	1.82	1.76E+05	3.04E-04	1.73	2.04E+05	2.66E-04	1.30
	2.27E+05	1.53E-04	0.674	1.88E+05	2.29E-04	1.22	1.98E+05	2.43E-04	1.23	1.75E+05	3.21E-04	1.83	1.74E+05	3.05E-04	1.75	2.01E+05	2.67E-04	1.33
VacW-215	2.24E+05	8.99E-05	0.401	1.99E+05	1.30E-04	0.653	2.00E+05	1.26E-04	0.632	1.91E+05	1.97E-04	1.03	2.06E+05	1.92E-04	0.936	2.05E+05	1.42E-04	0.695
	2.23E+05	9.11E-05	0.408	1.94E+05	1.50E-04	0.775	1.97E+05	1.44E-04	0.733	1.91E+05	1.93E-04	1.01	2.03E+05	2.02E-04	0.997	2.10E+05	1.38E-04	0.656

The data were shown from two independent experiments with similar results.
